# Identification and Characterization of *BTD* Gene Mutations in Jordanian Children with Biotinidase Deficiency

**DOI:** 10.3390/jpm10010004

**Published:** 2020-01-21

**Authors:** Laith N. AL-Eitan, Kifah Alqa’qa’, Wajdi Amayreh, Rame Khasawneh, Hanan Aljamal, Mamoon Al-Abed, Yazan Haddad, Tamara Rawashdeh, Zaher Jaradat, Hazem Haddad

**Affiliations:** 1 Department of Applied Biological Sciences, Jordan University of Science and Technology, Irbid 22110, Jordan; haaljamal139@sci.just.edu.jo; 2 Department of Biotechnology and Genetic Engineering, Jordan University of Science and Technology, Irbid 22110, Jordan; 3 Department of Pediatrics, Jordan University of Science and Technology, Irbid 22110, Jordan; kifah88@yahoo.com; 4 Department of Pediatrics, Metabolic Genetics Clinic, Queen Rania Al-Abdullah Children’s Hospital, King Hussein Medical Centre, Amman 11855, Jordan; wajdidr@yahoo.com; 5 Department of Pathology, Division of Molecular Genetic Pathology, King Hussein, Medical Center, Amman 11855, Jordan; rami.khasawneh@jaf.mil.jo; 6 Princess Haya Biotechnology Center, Jordan University of Science and Technology, Irbid 22110, Jordan; mmalabed@just.edu.jo (M.A.-A.); tarawashdeh@just.edu.jo (T.R.); zmjaradat@just.edu.jo (Z.J.); hazem_haddad1981@just.edu.jo (H.H.); 7 Department of Chemistry and Biochemistry, Mendel University in Brno, Zemedelska, 61300 Brno, Czech Republic; yazanhaddad@hotmail.com; 8 Central European Institute of Technology, Brno University of Technology, Purkynova, 61200 Brno, Czech Republic

**Keywords:** biotinidase deficiency, *BTD*, Jordan, enzyme assay, familial study, genetics

## Abstract

Biotinidase deficiency is an autosomal recessive metabolic disorder whose diagnosis currently depends on clinical symptoms and a biotinidase enzyme assay. This study aimed to investigate the mutational status and enzymatic activity of biotinidase deficiency in seven unrelated Jordanian families including 10 patients and 17 healthy family members. Amplified DNA was analyzed by the automated Sanger sequencing method, and the enzymatic assay was performed using a colorimetric assessment. Biotinidase level was significantly lower (*p* < 0.001) in *BTD* children compare to their non-affected family members. Genetic sequencing revealed six different mutations in Jordanian patients. One mutation was novel and located in exon 4, which could be a prevalent mutation for biotinidase deficiency in the Jordanian population. Identification of these common mutations and combing the enzymatic activity with genotypic data will help clinicians with regard to better genetic counseling and management through implementing prevention programs in the future.

## 1. Introduction

The biotinidase enzyme separates biotin from dietary protein-bound sources, and it is responsible for biotin recycling [1,2,3]. Biotinidase deficiency is an inherited autosomal recessive disorder where the biotinidase enzyme activity is blocked, resulting in the inability to release biotin from the diet and recycling this vitamin [1,2,4,5,6]. Symptoms of the disease begin to appear at the first 2–3 months of life or later throughout childhood [7,8]. The deficiency can be classified into profound and partial biotinidase deficiency based on the enzyme level measurement [1,9].

In profound biotinidase deficiency, some children may be asymptomatic until adolescence [10]. Many neurological and developmental symptoms can develop including seizures, learning disabilities, skin rash, hypotonia (decreased muscle tone), breathing problems, lack of coordination, hearing and vision loss, vision problems, mild hyperammonemia, alopecia (spot hair loss), behavioral disorders, developmental delay, and organic aciduria [2,10]. In partial biotinidase deficiency, untreated children may have any of the previously mentioned symptoms but at a milder level and depending on if the children are under stress situations [10]. Biotin supplements can reduce and even sometimes totally reverse the symptoms successfully with an appropriate dose of biotin [1,2,4,6].

The biotinidase enzyme is encoded by the *BTD* gene, which is located on chromosome 3p25 and contains four exons. The gene spans at least 44 kb and encodes a protein of 543 amino acids by exons 2 through 4 [1,2,3]. More than 165 mutations were identified in the *BTD* gene, whereby five of them account for approximately 60% of the mutant alleles in the general newborn screening for the deficiency [6,9]. Most of the mutations are either homozygous or compound heterozygous observed in all types of variants [5,6]. Currently, there is no or limited data about the frequency of these variants and their associations with biotinidase deficiency in the Jordanian population. Therefore, this study aimed to examine the genetic polymorphisms in the *BTD* gene and measure enzyme activity among affected Jordanian families.

## 2. Materials and Methods

### 2.1. Patient Recruitment

Having obtained ethical approval (no. 20/84/2015) by the ethics committee of the Institutional Review Board at Jordan University of Science and Technology, this study was conducted in accordance with the Declaration of Helsinki, 1964, as revised in 2013. All potentially eligible patients attending the Jordanian Royal Medical Services (RMS) were invited to participate. Parents of the patients were given a copy of the patient information sheet to read over and consider before participating in the study. Written informed consent was obtained only from those who agreed to participate in the study with three copies of the consent made for the patient, the patient’s medical record, and the research team.

### 2.2. Sample Collection

A family study conducted on 27 participants from seven unrelated consanguineous Jordanian families attended the genetic and metabolic clinic at RMS by collecting ethylenediaminetetraacetic acid (EDTA) blood samples. These families included 10 children diagnosed as biotinidase-deficient patients with an age range of 5.32 ± 5.09 (years ± SD).

### 2.3. DNA Extraction and Genotyping

Sample preparation and analysis were conducted at the Princess Haya Biotechnology Center (PHBC) at King Abdullah University Hospital (KAUH). Purification of the genomic DNA was performed using Gentra Puregene Blood Kits (Promega Corporation, Madison, WI, USA) in order to remove contaminants and inhibitors for large-volume samples. DNA concentration measurement was done using a Bio-Drop Spectrophotometer (Cambridge, UK), and quality criteria were defined at 50 ng/µL concentration with purity greater than 1.7 for the absorbance ratio of 260/280 nm. Polymerase chain reaction (PCR) was conducted using 10 pairs of primers to amplify the coding region of the biotinidase deficiency gene (Table 1). PCR products from each sample were run on 2% agarose gel electrophoresis and directly visualized using a gel documentation system (ChemiDoc; Bio-Rad Laboratories, Hercules, CA, USA). Amplified samples were subjected to direct automated sequencing using a 3130xl genetic analyzer (Applied Biosystems, Foster City, CA, USA). Data analysis was carried using ChromasPro software (Technolysium Pty Ltd., Queensland, Australia), where the base change was identified. Mutations were evaluated using Basic Local Alignment Tool (BLAST) and with reference to the longest complementary DNA (cDNA) transcript of the *BTD* gene, namely, transcript variant 1 (NM_001281723.3) in Genbank and BTD-213 (ENST00000643237.2) in the Ensembl database.

### 2.4. Enzyme Assay

The enzyme assay was performed using a colorimetric assay in punched filter paper discs. wherein biotinyl-*p*-aminobenzoate is the substrate as described previously by Heard et al. [11]. The enzymatic activity was assessed for all 27 participants as nmol/min/mL. An independent *t*-test for equality of means (equal variances not assumed) was used to compare the enzymatic activity of cases and their family members considering a *p*-value below 0.05 as significant.

## 3. Results

### 3.1. Clinical Characterization Data of BTD Patients and Mutation Analysis

Clinical characteristics related to the biotinidase deficiency were observed in all patients with variation in the associated neurological and dermatological traits (Table 2). None of the studied patients were mentally retarded or had any developmental delay. Only patients no. 3 and 7 had hearing loss, which is usually not reversible, in addition to conjunctivitis in patient no. 3 (Table 2). Among the seven included families, six of them were of consanguineous marriage.

**Figure 1:**
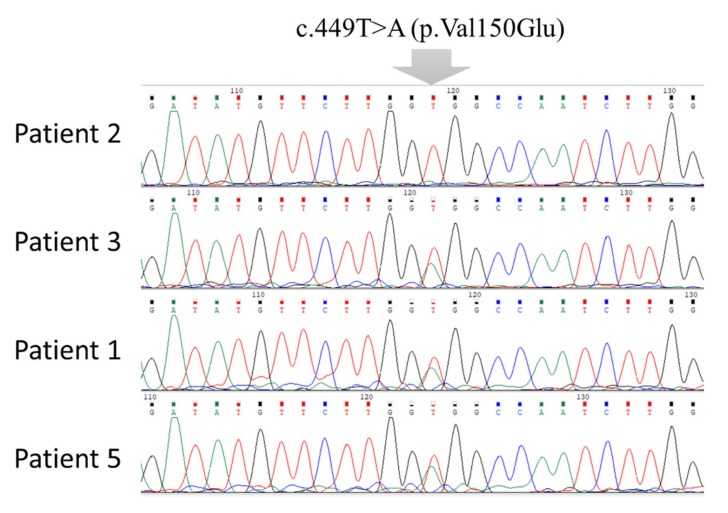
Representative chromatogram of the novel c.449T>A mutation.

The mutational screening revealed six different mutations, five of which were previously reported in the literature. These mutations are rs104893687, rs80338684, rs397514356, rs397514345, and rs397514411, in addition to a newly detected mutation in exon 4, c.449T>A (Table 3). In the patient of the second family that had three participants, the rs104893687 mutation was found in exon 2, which is a 235C>T missense variant resulting in a cysteine amino acid instead of arginine at position 79. This patient was also found to have another missense variant located in exon 4 (c.1301A>G, p.Tyr434Cys) resulting in a substitution of tyrosine at position 434 to cysteine. Family no. 6 showed an rs80338684 mutation in its three affected children. This 7-bp deletion/3-bp insertion in exon 3 (98-104del7ins3) caused a frameshift terminated with a stop codon that may result in a truncated protein. The rs397514411 mutation was another deletion/insertion variant located in exon 4 of the family no. 4 and 7. This single insertion resulted in a frameshift coding proline instead of leucine. The last reported mutation was a single deletion (rs397514356, p.Phe131Leu) in exon 3 of patients no. 1, 2, 4, and 6. The novel detected variant in our population was c.449T>A (p.Val150Glu), which was revealed in most patients (7/10), suggesting its importance (Figure 1).

### 3.2. Enzyme Activity

**Figure 2:**
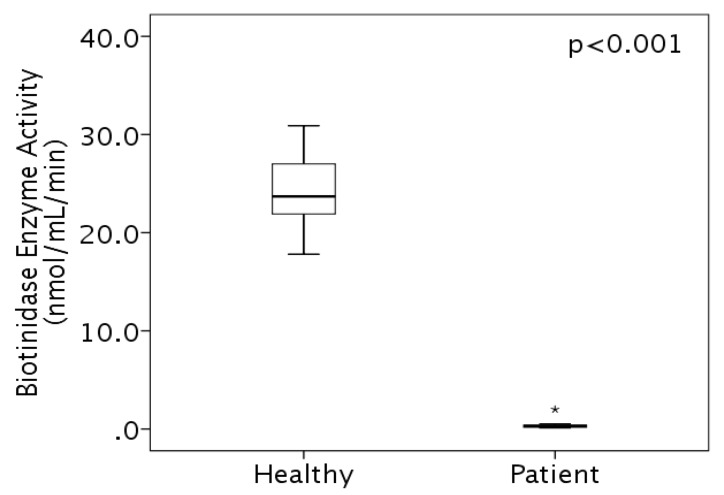
Biotinidase enzyme level in biotinidase gene (*BTD*)-deficient patients (*n* = 10) compared to their healthy family members (*n* = 17) measured as nmol·mL^−1^∙min^−1^. *: Outlier case.

Patients received pharmacologic doses of biotin at the time of sample collection to measure biotinidase activity. Enzyme activity was measured using a colorimetric assay and found to be 0.238 ± 0.142 (mean ± SD) in the affected children, which was significantly lower (*p* < 0.001) than the finding of 23.88 ± 3.62 (mean ± SD) in their healthy family members (Figure 2, Table 3).

## 4. Discussion

To the best of our knowledge, this study is the first of its kind among the Jordanian population to study the molecular and clinical status of biotinidase deficiency. The worldwide screening of biotinidase deficiency was assessed at one in 137,000 [12]. This disorder has a wide ethnic distribution, while most of the reported patients are of European descent [9]. Cutaneous manifestations including rash and alopecia were less commonly seen in our cohort (one of 10 and four of 10, respectively) consistent with previously published data on alopecia in Chinese patients [13]. In contrast, alopecia was found to be a common phenotype in Iranian and Indian patients (eight of 16 and nine of 10, respectively) [14,15]. Dermatological and neurological symptoms associated with the disease varied among the patients, and this could be due to the differences in the age of diagnosis and the age at biotin administration. Seizure is one of the common neurological symptoms associated with biotin deficiency, presented in seven patients in our cohort. Seizure was found to be one of the initial phenotypes associated with the disease with an occurrence of 70% in symptomatic children [10]. Biotin administration is highly recommended for children with poorly controlled seizures [10]. A preliminary study showed that patients with missense mutations appear to develop hearing loss, which seems to be irreversible even with a pharmacological dose (5–20 mg/day) of biotin treatment, especially if there is a delay in diagnosis and treatment [16,17]. On the other hand, hearing loss in children could be prevented by the intake of biotin immediately after birth [16]. Therefore, physicians should be aware of the symptoms at earlier ages, especially in families at risk, for better disease control and treatment outcomes.

Because of the increased incidence of consanguinity in Middle Eastern countries as in the case of Jordan, children were mostly homozygous or compound heterozygous for the mutations. Mutation analysis revealed five mutations that were previously observed in other populations, three of which were frameshifts resulting in the absence or shortening of the enzyme polypeptide chain. The deletion 393delC resulting in a frameshift encoding leucine instead of phenylalanine (F111L) was found in four children, two of whom were homozygous for the mutation and the other two of whom were heterozygous. According to Wolf et al., this mutation was found in two Palestinian and one Turkish child [18]. Another common frameshift variant, 1204insC (Leu402Pro), was homozygous in one child and heterozygous in another one. This mutation was also found in Spanish and Indian patients reported by Iqbal et al., using denaturing high-pressure liquid chromatography (dHPLC) [19]. Two children were homozygous for the 7-bp deletion (5′–GCGGCTG–3′)/3-bp insertion (5′–TCC–3′) (c.38_44delinsTCC/p.Cys13Phe), in addition to one heterozygous child. This mutation was homozygous in a Sri Lankan child, whereas it appears to be common in Western European countries, resulting in the synthesis of truncated inactive enzyme protein [20]. Ten patients with this deletion/insertion mutation from a different ethnic background and a non-consanguineous marriage were found to have a biotinidase activity range of 0.0 to 0.7 nmol/min/mL [21]. The other two mutations found in exon 2 and 4 were missense, resulting in coding a different amino acid in the encoded enzyme protein. The p.Tyr434Cys variant was detected only in one patient (no. 3), and it might interfere with the normal disulfide bonding, causing its aberrant formation. This is consistent with Wolf et al.’s study conducted in the United States of America (USA), whereas one patient was found to have a c.1361A>C (p.Tyr454Cys) mutation with a complete absence of the biotinidase activity (0.0 nmol/min/mL) [22]. The last detected mutation in our cohort was c.235C>T (p.Arg79Cys) in exon 2, which is common in different populations from South China [23], Iran [24], Poland [25], Turkey [17,26,27], United States [22], and Sweden [28]. The Iranian, Turkish, and American children were found to have an enzyme activity of 0.11, 0.13, and 0.0 nmol/min/mL, respectively [22,24,26]. Asgari et al. measured the enzyme activity in eight of their patients, and the activity was in a range of 1.09 ± 1.37 nmol/min/mL [24], which is much higher than our reported values (0.238 ± 0.142). Studies found that patients with a complete absence of biotinidase activity are more likely to develop symptoms than individuals with some enzymatic activity [12]. The complete absence of enzyme activity is usually a result of deletion, insertion, or nonsense mutations, whereas it is not certain in the case of missense mutations [12]. The novel mutation c.449T>A leads to a new reading frame due to the change of codon valine 170 to glutamic acid, and it is reported for the first time with a high frequency in patients compared to the other detected mutations, which suggests that it could be a founder mutation in the Jordanian population, where it could help in disease diagnosis and assessment. Identification of local mutations is very important for development of effective newborn screening. A recent report from the Czech Republic showed successful confirmed diagnosis of 21 *BTD* patients out of 181,396 screened neonates in the period from June 2017 to end of 2017 [29]. In Minas Gerais state in Brazil, a newborn screening program tested over one million neonates for *BTD* since it was introduced to the program in 2013, with novel variants identified in 14 patients [30].

Recently, Borsatto et al. reported in vitro confirmation of the effect of *BTD* gene mutations on enzyme activity [31]. They highlighted the deleterious consequences of Leu40Pro, Cys160Tyr, and Leu446Pro variants. The researchers previously reported 39 variants in the *BTD* gene in a cohort of Brazilian patients [32]. The most frequent pathogenic variants were c.1330G>C, c.755A>G, and c.[1330G>C;511G>A].

## 5. Conclusions

This study emphasizes the importance of including *BTD* gene sequencing and biotinidase activity measurement in the neonatal screening program, especially in families at risk. This measurement should take into account the high rate of endogamy in our region affecting the incidence of autosomal disorders, for earlier diagnosis and treatment of the inherited deficiency to overcome the consequences of late diagnosis and to lower the healthcare burdens. In addition, as not many pharmacogenetic and individualized medicine studies [33,34] were conducted in Jordan, genotype–phenotype correlations need further and more precise analysis and establishment, since the same mutation could result in various phenotypes due to the differences in genetic background and environmental factors.

## Figures and Tables

**Table 1:** Sequence and product size of the PCR primers used for DNA amplification.

Exon No. ^†^	Forward Primer	Reverse Primer	Product Size
1	AGAATGTAAACACGCGCGTT	AGAGCGTAAACCACAAAGCG	465 bp
2	TCTTTGAGCCGCAGTATCAC	TTCAGAGGGTGGTAGGAAGC	554 bp
3	ATGAATGCAGCGGTTCTTCC	TGGCACATGGATCTTTGGGA	360 bp
4a	GGTGGTCTCAATCTCCTGAC	GTGGAGATAGCCTTCCTTTC	892 bp
4b	GCGATCCGTACTGTGAGAAG	AGACCAATCGCATACTGAGAGA	818 bp

**^†^** Two overlapping fragments of exon 4 (a and b).

**Table 2:** Clinical characteristics of 10 biotinidase gene (*BTD*)-deficient patients in Jordan.

Family No.	Patient No.	Gender ^†^	Age of Diagnosis	Age at Biotin Administration	Consanguinity	Neurological Traits ^‡^							Dermatological Traits	
						Seizures	Hypotonia	Ataxia	Speech Delay	Hearing Loss	Mental Retardation	Conjunctivitis	Alopecia	Rash
1	1	M	2 weeks	Since birth	Yes	No	No	No	No	No	No	No	No	No
	2	F	6 months	6 months		Yes	Yes	No	No	No	No	No	No	No
2	3	M	3 months	3 months	Yes	Yes	Yes	No	Yes	Yes	No	No	Yes	No
3	4	F	27 months	27 months	Yes	Yes	Yes	Yes	Yes	No	No	Yes	Yes	Yes
4	5	F	8 months	9 months	Yes	Yes	Yes	NA	NA	No	No	No	No	No
5	6	F	21 months	21 months	Yes	Yes	Yes	Yes	No	No	No	No	Yes	No
6	7	M	12 months	4 months	Far relatives	Yes	Yes	No	No	Yes	No	No	No	No
	8	M	4 months	3 months		No	No	No	No	No	No	No	No	No
	9	M	9 months	3.5 months		No	No	No	No	No	No	No	No	No
7	10	F	3 years	3 years	Yes	Yes	Yes	Yes	Yes	No	No	No	Yes	No

**^†^** M: male; F: female. **^‡^** NA: Not available.

**Table 3:** *BTD* gene genotyping and enzyme activity measurement in biotinidase-deficient patients.

Family No.	Patient No.	Exon	Mutation	Nucleotide Change ^†^	Variant Type	Protein Change	Enzyme Assay (nmol/min/mL)
1	1	3	rs397514356	del C hetero	Frameshift	Phe111Leu	0.2
		4	c.449T>A	T/A	Missense	Val150Glu	
	2	3	rs397514356	del C hetero	Frameshift	Phe111Leu	0.3
		4	c.449T>A	T/A	Missense	Val150Glu	
2	3	2	rs104893687	C/T	Missense	Arg79Cys	0.3
		4	c.449T>A	T/A	Missense	Val150Glu	
		4	rs397514345	A/G	Missense	Tyr434Cys	
3	4	3	rs397514356	del C homo	Frameshift	Phe111Leu	0.5
4	5	4	c.449T>A	T/A	Missense	Val150Glu	0.17
		4	rs397514411	Ins C hetero	Frameshift	Leu402Pro	
5	6	3	rs397514356	del C homo	Frameshift	Phe111Leu	0.3
6	7	2	rs80338684	del/ins homo GCGGCTG/TCC	Frameshift	Cys13Phe	0.4
		4	c.449T>A	T/A	Missense	Val150Glu	
	8	2	rs80338684	del/ins hetero GCGGCTG/TCC	Frameshift	Cys13Phe	0.1
		4	c.449T>A	T/A	Missense	Val150Glu	
	9	2	rs80338684	del/ins homo GCGGCTG/TCC	Frameshift	Cys13Phe	2.0
		4	c.449T>A	T/A	Missense	Val150Glu	
7	10	4	rs397514411	Ins C homo	Frameshift	Leu402Pro	0.1

**^†^** Del: deletion; Ins: insertion; Homo: homozygous; Hetero: heterozygous.
